# Marker-assisted selection complements phenotypic screening at seedling stage to identify cassava mosaic disease-resistant genotypes in African cassava populations

**DOI:** 10.1038/s41598-021-82360-8

**Published:** 2021-02-02

**Authors:** Bunmi Olasanmi, Martina Kyallo, Nasser Yao

**Affiliations:** 1 Department of Agronomy, University of Ibadan, Ibadan, Nigeria; 2 Bioscience eastern and central Africa-International Livestock Research Institute (BecA-ILRI) Hub, Nairobi, Kenya; 3 Alliance Bioversity International-CIAT, CIAT Africa Office, Nairobi, Kenya

**Keywords:** Genetics, Plant sciences

## Abstract

Cassava mosaic disease (CMD) is a serious threat to cassava production in sub-Saharan Africa. The use of genomic-assisted selection at the seedling trial stage would help to reduce the time for release, breeding cost, and resources used, hence increase selection efficiency in cassava breeding programs. Five cassava populations were screened for resistance to CMD during the seedling evaluation trial at 1, 3, and 5 months after planting using a scale of 1–5. The genotypes in the five populations were also screened using six molecular markers linked to the CMD2 gene. The correlation between the phenotypic and marker data was estimated. Based on Cassava Mosaic Disease Severity Score (CMDSS), between 53 and 82% of the progenies were resistant across the populations with an average of 70.5%. About 70% of the progenies were identified to be resistant to the disease across the populations with a range of 62–80% using the marker data. With both marker data and CMDSS combined, 40–60% of the progenies in each population, with an average of 52%, were identified to be resistant to CMD. There was a fairly significant correlation between the marker data and CMDSS in each cassava population with correlation coefficients ranging from 0.2024 to 0.3460 suggesting that novel genes not associated to the markers used might be involved in the resistance to CMD. The resistant genotypes identified in this study with potential for other desirable traits were selected for evaluation at the advanced trial stage thereby shortening the period required for the breeding program.

## Introduction

Cassava (*Manihot esculenta* Crantz) is an important subsistence and food security crop for resource-poor households in about 40 African countries where it is a mainstay of over 200 million people^1–5^. It is said to be the fourth most important source of food calories for humans in the tropics^6^. Globally, Nigeria is the leading producer of cassava roots with production estimated at 59.5 million metric tonnes^7^. In Nigeria, cassava is ranked among the major food crops supplying 70% of the total calorie intake of about half of the population^8^. However, CMD, a devastating and debilitating disease caused by cassava mosaic begomoviruses (CMBs) is constraining cassava cultivation in all cassava growing areas^5,9–12^. Some of the improved cassava varieties currently cultivated in Nigeria are susceptible to CMD. It was reported that African cassava mosaic virus (ACMV) greatly decreased the growth and yield of susceptible varieties^13,14^. Strategies for controlling the disease include the use of resistant varieties^15–18^.

The resistance to CMD is known to be polygenic; the International Center for Tropical Agriculture (CIAT) has mapped two CMD resistance genes namely CMD1 (recessive gene) and CMD2 (major dominant gene)^11,19^ and three molecular markers associated with CMD2, namely RME1, SSRY28, and NS158, were developed^20^. These markers are very useful and hold great promise in fast-tracking improvement of cassava for CMD-resistance^21^. The molecular markers were used by Bi et al. ^21^ to screen some varieties of cassava for resistance to CMD. In addition to these markers (RME1, NS158, and SSRY28), markers SSRY106, NS169, and NS198 have been used in various breeding programs to screen for resistance to CMD^22–24^.

The conventional methods used for selection in breeding programs are always slow and unreliable. Obtaining reliable phenotypic data for complex traits is especially difficult and is often the biggest bottleneck to the eventual application of MAS^25,26^. There is a need for clonal multiplication of new genotypes to ensure proper phenotypic evaluation and this may require 4–5 years in conventional breeding because of the low multiplication ratio in cassava^27^. According to Xu and Crouch^28^, some of the main applications of molecular marker technologies in crop breeding include breeding for traits difficult to improve through conventional phenotypic selection because they are expensive or time-consuming to measure. They also stated that traits whose selection depends on specific environments or developmental stages for expression of the target phenotype could be improved using the marker technologies. Screening for CMD using conventional methods could be unreliable if the genotypes are not assessed for incidence and severity at the peak of the disease incidence in the locality. The stage of development of the cassava genotypes at the time of assessment for CMD could lead to wrong selection. Also, data collected in a season is not reliable enough to select CMD resistant cassava genotypes due to the influence of environment and seasonal variation on severity and incidence of CMD.

Five cassava populations were developed at University of Ibadan, Nigeria in 2016 for the improvement of cassava for beta-carotene content, CMD resistance, plant architecture, and other desirable traits. To fast-track and increase precision in the improvement of cassava for important traits, there is a need to complement phenotypic data collected at the early breeding stage with screening using molecular markers associated with such traits. Therefore, the objective of this study was to screen newly developed cassava genotypes in the five populations for CMD resistance using six SSR markers associated with CMD resistance and phenotypic data collected in one season at the seedling evaluation stage.

## Materials and methods

### Source of plant materials and phenotype screening for CMD resistance

Six hundred and five genotypes from five open-pollinated cassava populations involving five female parents in an ongoing breeding research program at the University of Ibadan Nigeria for improvement of cassava for CMD resistance, beta-carotene content, and plant architecture were used for this study (Table 1). The seeds were generated in the 2016/17 growing season and sowed in the nursery in March 2017 on nursery beds at the Research field of Department of Agronomy, University of Ibadan. The seedlings were transplanted to the field seven weeks after sowing and the plants were watered for the first two weeks to aid their establishment due to the dry spell at that period. The field evaluation was done in an uncontrolled environment and the plants were only exposed to a natural source of inoculum. Whitefly (*Bemisia tabaci*) which is the vector for CMBs was observed in the field throughout the evaluation of the plants for CMD.

**Table 1:** The cassava populations and the check varieties used for this study.

Pop	Female parent	Response to CMD	Number of genotypes/pop
1	IITA-TMS-I070593	Resistant	153
2	IITA-TMS-I011371	Tolerant	100
3	IITA-TMS-I070539	Resistant	151
4	IITA-TMS-I011368	Tolerant	100
5	IITA-TMS-I011412	Tolerant	101
**Total**			**605**
*Check varieties*			
1	IITA-TMS-97-2205	Resistant	
2	IITA-TMS-30572	Tolerant	
3	IITA-TMS-30555	Tolerant	
4	TME 3	Resistant	

**Plate 1:**
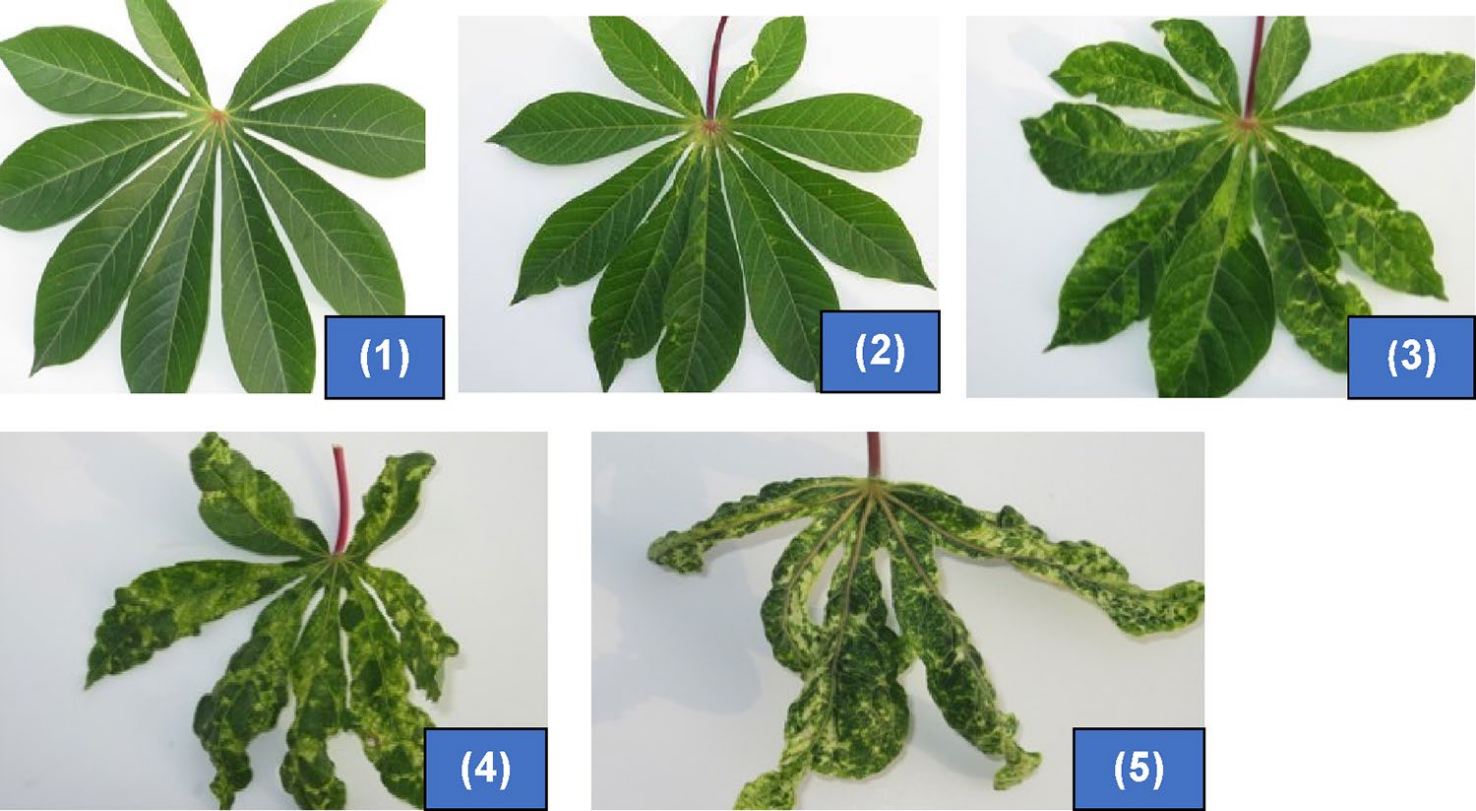
Scale used in scoring for cassava mosaic disease^1,29^. 1 = No visible symptoms (highly resistant). 2 = Mild chlorotic patterns (moderately resistant). 3 = Mosaic patterns on all leaves and leaf distortion (mildly susceptible). 4 = Mosaic pattern on all leaves, leaf distortion, and a general reduction in leaf size (susceptible). 5 = Misshapen and twisted leaves and stunting of the whole plant (highly susceptible).

All the cassava genotypes (progenies, parents, and checks) screened in this study were evaluated for CMD severity at 1, 3, and 5 months after planting (MAP) using the 1–5 scale where 1 represents no symptom expression and 5, severe symptom expression^29^ (Plate 1). The genotypes were screened at three stages (1, 3, and 5 MAP) in the life cycle of the plants to ensure the susceptible genotypes without symptoms at a stage are detected at another stage(s). The maximum CMD severity score (CMDSS) recorded at any of the three stages was used to classify each genotype. The selection of genotypes in each population for molecular screening was done to include at least one progeny for each of the CMDSS 2, 3, 4, and 5 while others had CMDSS of 1.

### Sample collection and DNA extraction

About 10 g of young leaves were stored in a labeled zip-lock bag packaged with silica gel to dry the leaves. As a backup, some leaf samples for each genotype/variety were also collected in labeled paper envelopes and oven-dried at 48 °C for about 48 h. The dry leaves in paper envelopes were packed in big zip-lock bags with silica gel to avoid the absorption of moisture. All the leaf samples were shipped to the BecA-ILRI Hub, Nairobi in September 2017 for molecular screening.

Total DNA was extracted from approximately 150 mg silica gel dried leaf tissue using a ZR-96 Plant/Seed DNA kit (Zymo Research Corp.) with slight modification whereby 10% dithiothreitol (DTT) was used in place of beta-mercaptoethanol and the extracted genomic DNA was eluted twice using 50 µl elution buffer each time. The extracted DNA was analyzed by electrophoresis on a 0.8% agarose gel and the concentration and purity were determined using a NanoDrop 2000C spectrophotometer (Thermo Fischer Scientific).

### CMD resistance screening by PCR and capillary electrophoresis

Six molecular markers (Table 2) associated with the CMD2 gene used in previous studies^22–24,30,31^ were selected to screen the cassava genotypes for CMD resistance. Multiplex PCRs were run after determining the working annealing temperature which ranged from 50 to 65 °C for each primer using gradient PCR. The product size and dye color of the primers were considered in forming the multiplex groups. The PCR mix of the final volume of 20 µL contained AccuPower PCR PreMix without dye (Bioneer, Korea), 0.1–0.2 pM of each primer (Table 2), 30 ng genomic DNA, 0.5 mM additional MgCl_2,_ and nuclease-free water. Amplification was performed in a GeneAmp PCR System 9700 thermocycler (Applied Biosystems, Foster City, CA) using the following PCR program: initial denaturation at 94 °C for 3 min; followed by 35 cycles at 94 °C for 30 s, 55 °C for 1 min, and 72 °C for 2 min; and a final extension at 72 °C for 10 min. The multiplex products were size fragmented in a 1.5% agarose gel stained with 0.25× GelRed (Biotium, USA) and run at 7 V/cm in 0.5× Tris TBE buffer. The gels were visualized under UV light using the UVP GelDoc-It Imaging System.

**Table 2:** CMD resistance linked SSR markers used in the study along with their estimated amplicon size.

S/N	Marker	Multiplex group	Dye color	Final concentration (pM/µl)	Estimated amplicon size (bp)	Source
1	NS 158	CL2	VIC (Green)	0.040	166	Okogbenin et al.^24^
2	NS 169	CL2	NED (Yellow)	0.075	319	Okogbenin et al.^24^
3	NS 198	CL2	PET (Red)	0.075	196	Okogbenin et al.^24^
4	RME 1	–	NED (Yellow)	0.090	700	Okogbenin et al.^24^
5	SSRY 106	CL1	VIC (Green)	0.090	270	Lokko et al.^22^
6	SSRY 28	CL1	NED (Yellow)	0.099	180	Lokko et al.^22^

The amplified PCR products were prepared for capillary electrophoresis by mixing 0.7–1.5 µl of each PCR product, depending on their concentration, with 9 µl of HIDI formamide (Applied Biosystems, USA) and 1 µl of GeneScan 500 LIZ Size Standard (Applied Biosystems, USA). The mixture was then denatured at 95 °C for 3 min followed by snap-chilling on ice-water for 5 min to prevent the denatured DNA from re-annealing. The fragments were analyzed by capillary electrophoresis on a Genetic Analyzer 3730 (Applied Biosystems, USA) at the BecA-ILRI hub in Nairobi, Kenya.

### Data analysis

The alleles were sized using the GeneMapper version 4.1 (Applied Biosystems, USA). The microsatellite data (allele size) for all the loci were subjected to allele frequency analysis using PowerMarker software V3.25. The phenotypic data (CMD severity scores—CMDSS) were subjected to descriptive analysis (mean and plotting of bar charts) using Microsoft Excel Software. Correlation between the phenotypic and discriminating marker data was estimated using Statistical Analysis System (SAS) Software Version 9.0^32^. The markers found to be polymorphic between the resistant and susceptible checks and at the same time discriminating among the progenies were used to select resistant progenies in each population. Progenies identified as resistant by both the marker and phenotypic scoring were selected as CMD resistant in this study.

The ability of the markers used in this study to predict the response of genotypes to CMD (resistance or susceptibility) was assessed by computing the accuracy (ACC) which is the proportion of correctly predicted genotypes, either as resistant or susceptible; the false-positive rate (FPR) which is the proportion of genotypes predicted to be resistant but were diseased also referred to as type I error; and the false-negative rate (FNR) which is the proportion of genotypes predicted to be susceptible but were resistant or type II error. The estimates were made using the formula below:

$$\begin{aligned} &{\text{Accuracy}}\;\left( {{\text{ACC}}} \right) = \left( {{\text{TP}} + {\text{TN}}} \right)/\left( {{\text{TP}} + {\text{FN}} + {\text{FP}} + {\text{TN}}} \right) \\ & {\text{False-positive rate}}\left( {{\text{FPR}}} \right) = {\text{FP}}/\left( {{\text{FP}} + {\text{TN}}} \right) \\ & {\text{False-negative rate}}\left( {{\text{FNR}}} \right) = {\text{FN}}/\left( {{\text{FN}} + {\text{TP}}} \right) \\ \end{aligned}$$

TP = True positive; FP = False positive; TN = True negative; FN = False negative.

## Results

### CMD severity scores for cassava genotypes evaluated in Ibadan in 2017

**Figure 1:**
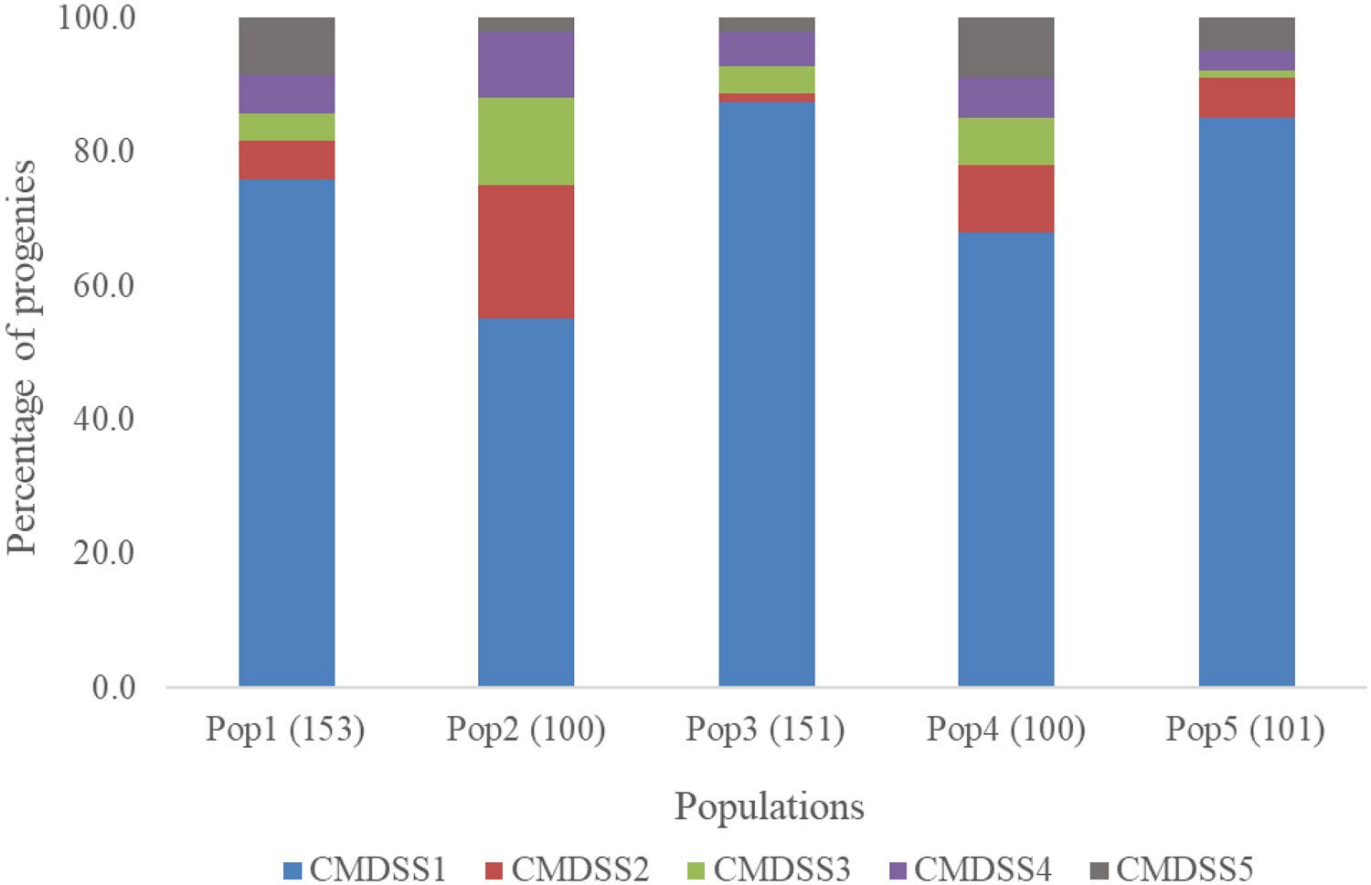
Cassava Mosaic Disease Severity Scores (CMDSS) of progenies in five cassava populations evaluated in Ibadan, Nigeria in 2017 (progeny size in parenthesis).

The CMDSS of the progenies in each cassava population screened in this study are shown in Fig. 1. About 76% (457) of the progenies across the five populations were highly resistant to CMD (genotypes with CMD severity score of 1) while 8% (47), 5% (33), 6% (36), and 5% (32) had CMDSS of 2 (moderately resistant), 3 (mildly susceptible), 4 (susceptible) and 5 (highly susceptible), respectively. About 76% or more of the progenies in populations 1, 3, and 5 had a severity score of 1 while only 55 and 68% of the progenies in populations 2 and 4, respectively, had CMDSS of 1.

### Informativeness of selected SSR markers

Marker RME was excluded in the final screening of the genotypes because the capillary electrophoresis could not analyze fragment sizes larger than 500 bp. The major allele frequency among the markers ranged from 0.33 (SSRY106) to 0.86 (NS 158) with an average of 0.62 while the number of genotypes (based on the discrimination among the progenies by each marker) ranged from 7 to 15 with an average of 11.2 (Table 3). The five SSR markers used in this study produced a total of 25 alleles. The number of alleles per marker ranged between 4 and 6 with an average of 5 alleles per marker.

**Table 3:** Allelic diversity parameters for markers linked to CMD resistance.

Marker	Major allele frequency	Number of genotype	Number of Alleles	Gene diversity	Heterozygosity	PIC
SSRY028	0.68	15	6	0.5011	0.4209	0.4719
SSRY106	0.33	15	6	0.7576	0.6248	0.7165
NS169	0.75	11	5	0.4053	0.3639	0.363
NS 198	0.49	8	4	0.6257	0.5673	0.5547
NS 158	0.86	7	4	0.2471	0.1311	0.2256
Mean	0.62	11.2	5	0.5074	0.4216	0.4664

*PIC* polymorphism information content.

The gene diversity, level of heterozygosity, and polymorphism information content (PIC) followed the same pattern among the markers. Markers with high PIC revealed high gene diversity and heterozygosity. Marker SSRY 106 had the highest value for each of the three parameters while NS 158 had the least value for each. Markers SSRY 028 and SSRY 106 had the same number of genotypes (15) though the latter had higher values for PIC, heterozygosity, and gene diversity. Consequently, the markers with high major allele frequencies had low values for PIC, heterozygosity, and gene diversity.

### Correlation between phenotypic and marker data in five open-pollinated cassava populations

There was a moderate correlation between the marker data and CMDSS in each of the five cassava populations with correlation coefficients ranging from 0.2024 (population 2) to 0.3460 (population 4) (Table 4). Based on CMDSS, between 53 and 82% of the progenies were CMD resistant across the five populations with an average of 70.4% (Table 5). Approximately 70% of the progenies were also identified to be CMD resistant across the five populations with a range of 62–80% using the marker data. With the marker data and CMDSS combined, 40–60% of the progenies were identified to be CMD resistant with an average of 52.4% across the five populations. Between 8 and 40 genotypes classified as resistant based on CMDSS were not confirmed so by genetic marker data while 9–28 genotypes classified as resistant by marker data were susceptible based on phenotypic data (CMDSS). The rate of misclassification ranged between 26.4 and 39.0% across the five populations while the level of accuracy ranged between 0.61 and 0.74 (Table 6). The false-positive rate ranged from 0.47 to 0.59 while the false-negative rate ranged from 0.11 to 0.30 among the populations (Table 6).

**Table 4:** Correlation coefficients between marker data and CMDSS in five cassava populations developed in Nigeria.

Population	Female parent	Number of progenies	Correlation coefficient (*p* value)
1	IITA-TMS-I070593	153	0.2222 (0.0066)
2	IITA-TMS-I011371	100	0.2024 (0.0384)
3	IITA-TMS-I070539	151	0.2056 (0.0111)
4	IITA-TMS-I011368	100	0.3460 (0.0003)
5	IITA-TMS-I011412	101	0.2116 (0.0355)

**Table 5:** Proportion of CMD resistant individuals identified in five cassava populations using phenotypic and marker data along with the corresponding number of genotypes.

Pop	Female parent	Number of progenies	CMDSS (%)	Marker data (%)	Number of genotypes classified resistant by CMDSS but not by marker data (%)	Number of genotypes classified resistant by marker data but not by CMDSS (%)	Marker data and CMDSS (%)
1	IITA-TMS-I070593	153	75 (115)	63 (97)	29 (19)	18 (12)	52 (79)
2	IITA-TMS-I011371	100	53 (53)	66 (66)	13 (13)	28 (28)	40 (40)
3	IITA-TMS-I070539	151	82 (124)	63 (95)	40 (26)	11 (7)	56 (84)
4	IITA-TMS-I011368	100	68 (68)	80 (80)	8 (8)	20 (20)	60 (60)
5	IITA-TMS-I011412	101	74 (75)	62 (63)	22 (22)	9 (9)	54 (54)
Mean		121	70.4 (87)	66.8 (80.2)	22.4	17.2	52.4 (63.4)

**Table 6:** Confusion matrix for the polymorphic markers used to screen five cassava populations for resistance to cassava mosaic disease.

Pop	Discriminating marker	Prediction	Truth		Misclassification (%)	Accuracy	False-positive rate	False-negative rate
			Resistant	Susceptible				
1	SSRY 106	Resistant	83	18	31.8	0.68	0.50	0.26
		Susceptible	29	18				
2	NS 158	Resistant	44	28	39.0	0.61	0.58	0.23
		Susceptible	13	20				
3	SSRY 106	Resistant	88	11	33.8	0.66	0.48	0.31
		Susceptible	40	12				
4	SSRY 106	Resistant	64	20	26.4	0.74	0.59	0.11
		Susceptible	8	14				
5	SSRY 106	Resistant	58	9	31.3	0.69	0.47	0.28
		Susceptible	22	10				

## Discussion

The observed high number of resistant genotypes in the five populations found in this study is due to the consideration given to CMD resistance during the selection of genotypes for molecular screening, hence, the result is not a reflection of the level of segregation for CMD in each population. Some of the genotypes characterized to be resistant at the early growth stage were later found to be susceptible resulting in about 25% of the genotypes being susceptible. It has been suggested that the increased severity in some genotypes at later stages in the breeding scheme could be a result of the accumulation of virus in planting materials, as cassava is normally vegetatively propagated^23^. This, therefore, calls for thorough screening of cassava genotypes for their response to CMD across seasons and locations where molecular screening is impossible to ensure that selected genotypes are certified CMD-resistant.

The number of alleles at a determined SSR locus (allelic richness) is the simplest measure of genetic diversity^33^. The allelic richness per locus which varied among the markers from 4 to 6 (with an average of 5) observed in this study indicates high polymorphism of the selected SSR markers resulting in high display of the genetic diversity among the progenies in each population relative to CMD resistance. This, therefore, provides ample opportunity for selection for CMD resistance coded for by the locus the markers are associated with among the genotypes in the cassava populations. The close range of 4–6 alleles per locus among the markers corroborates the fact that the markers are linked to the same gene^11,19^. However, the observed situation of 9–28 genotypes (depending on the population) being resistant by marker data but not confirmed by the phenotypic screening calls for reflection on the type of genetic mechanism and/or action involved in resistance to CMD. Gene pyramiding involving CMD 2 and other CMD resistant genes may therefore be needed to confer stronger resistance to CMD in the region.

The high PIC, gene diversity and heterozygosity observed for most markers indicate a high level of genetic diversity for CMD resistance in the cassava populations regardless of the number of markers linked to the same gene used in this study. Polymorphism information content (PIC) is the measure used to calculate the discrimination power and informativeness of SSR markers^34^, hence, PIC value is a measure of polymorphism among genotypes for a marker locus used in genetic diversity analysis since it reflects allelic diversity and frequency among the genotypes^35^. The PIC can be classified as satisfactory (PIC > 0.5), medium (0.25 ≤ *P* ≤ 0.5) and low (PIC < 0.25)^34^ and markers with PIC values exceeding 0.5 are very efficient in discriminating genotypes and extremely useful in detecting the polymorphism rate at a particular locus^36^. In our study, two markers (SSRY106 and NS 198) had PIC values that exceeded 0.5 and were most useful in discriminating among the genotypes in the five populations for CMD resistance. However, the remaining markers with PIC values in the medium range were also useful in screening the populations for CMD resistance; thereby complementing the two markers with high PIC and the phenotypic data. It is noteworthy that the PIC values (0.2256–0.7165) observed for the SSR markers used in this study are higher than the values (0.049–0.375) reported in a study where 105 cassava landraces were assayed with 195 SNP markers^37^. The higher PIC values observed in this study is due to the multiallelic nature of SSRs compared to SNPs which are bi-allelic and can only have PIC values between 0.000 and 0.500^37^. However, the range observed for the SSRs used in this study is consistent with values (0.030–0.780) reported in the past study where 89 accessions of cassava were screened using 35 SSR markers^38^ despite the difference in the number of markers used, the population size and the type of alleles concerned. The similarity in the PIC values could suggest broader use of the SSR markers used in this study for general diversity study and population structure analysis without the focus on screening for CMD resistance.

The moderate correlation observed between the marker data and CMDSS in the five cassava populations screened in this study may be because only the CMDSS data collected during the first-year evaluation of the genotypes using a single plant per genotype (seedling nursery) were used. Earlier studies on screening cassava genotypes for CMD resistance were carried out over many seasons to ensure the reliability of the data^22,23^. Therefore, field screening of the genotypes used in this study over years using many vegetative propagules in replicated trials at advanced breeding stages and possibly across locations may increase the correlation coefficient between the field scores and the marker data thereby increasing the precision of the markers with the field scores. However, the use of markers at this early stage using one-year field screening data helps reduce the cost of such field evaluations and fast track the breeding efforts. Also, earlier studies have shown variation in the consistency of the markers used in this study^30,39^, hence, the use of the marker data alone may not be exceptionally reliable. However, a combination of the phenotypic and marker data in this study increased the precision of identifying CMD resistant genotypes thereby reducing the rigours of evaluating the genotypes over seasons and across locations.

The high level of disparity in the number of genotypes identified as resistant by CMDSS and marker data as shown by the rate of misclassification, level of accuracy, false-positive and false-negative rates in this study has implications in relation to the genetics of resistance to CMD among the progenies in the five populations as well as strains of the cassava mosaic virus in the area where the cassava genotypes were evaluated. A new source of CMD resistance was reported in the populations studied in the past^24^. The genotypes classified as resistant to CMD by phenotypic data only in this study may also have additional sources of resistance to the disease other than the CMD2 gene the markers are associated with, hence, there may be a need to screen the populations further for possible new sources of resistance to CMD. Also, those classified as resistant by markers only but were susceptible based on phenotypic data suggest there may be other strains of cassava mosaic virus in the research environment against which the CMD2 gene cannot confer resistance. This, therefore, calls for further investigation to ascertain if there are new sources of CMD resistance in such cassava genotypes not classified as resistant by the markers. We also agree with earlier submission that this may provide a solution to one of the major challenges in cassava breeding which is how to overcome the evolutionary capacity of the disease^24^. The additional sources of resistance to the disease are critical in building durable and stable resistance to CMD through gene pyramiding^24,40^. There is also a need for a survey of the research area for existing cassava mosaic virus strains. This will help to ascertain the strains of the virus causing the disease in the region.

## Conclusion

We were able to reduce the time needed to screen five new cassava populations for CMD resistance from at least two years of replicated trials across locations before selection when using the conventional method to less than a year by using molecular markers and phenotypic data. This study has therefore shown once again that marker-assisted selection is a powerful tool for fast-tracking cassava breeding programs. However, considering the moderate significance of the correlation between the field evaluation scores and the marker data, the use of both methods for selection of resistant genotypes to be evaluated for other traits of interest at advanced breeding stages made before harvesting of the seedling trial would increase the reliability of the selection. Therefore, in this study, markers were considered alongside the CMDSS to select the resistant genotypes to ensure higher precision. However, the level of inconsistency between the CMDSS and marker data calls for further studies on the possible existence of new cassava mosaic virus strains in the research area and likely additional sources of CMD resistance in the populations or genes interfering in combination to provide resistance. The high level of genetic variability revealed by these markers also calls for their investigation for broader genetic diversity study and population structure analysis without reference to the allele sizes for CMD resistance.
